# Severity of DSS-induced colitis is reduced in Ido1-deficient mice with down-regulation of TLR-MyD88-NF-kB transcriptional networks

**DOI:** 10.1038/srep17305

**Published:** 2015-11-27

**Authors:** Woo-Jeong Shon, Young-Kwan Lee, Ji Hee Shin, Eun Young Choi, Dong-Mi Shin

**Affiliations:** 1 Department of Food and Nutrition, Seoul National University, Seoul 151-742, Korea; 2 Department of Biomedical Sciences, Seoul National University College of Medicine, Seoul 110-799, Korea; 3 Research institution of human ecology, Seoul National University, Seoul 151-742, Korea

## Abstract

Indoleamine 2,3 -dioxygenase 1 (IDO1) catalyzes L-tryptophan to kynurenine in the first and rate-limiting step of tryptophan metabolism. IDO1 is expressed widely throughout the body, with especially high expression in colonic intestinal tissues. To examine the role of IDO1 in the colon, transcriptome analysis was performed in both *Ido1*^−/−^ and *Ido1*^+/+^ mice. Gene set enrichment analysis identified the Inflammatory Response as the most significant category modulated by the absence of IDO1. This observation prompted us to further investigate the function of IDO1 in the development of tissue inflammation. By using DSS-induced experimental colitis mice models, we found that the disease in *Ido1*^−/−^ mice was less severe than in *Ido1*^+/+^ mice. Pharmacological inhibition of IDO1 by L-1MT attenuated the severity of DSS-colitis as well. Transcriptome analyses revealed that pathways involving TLR and NF-kB signaling were significantly down-regulated by the absence of IDO1. Furthermore, dramatic changes in TLR and NF-kB signaling resulted in substantial changes in the expression of many inflammatory cytokines and chemokines. Numbers of inflammatory cells in colon and peripheral blood were reduced in IDO1 deficiency. These findings suggest that IDO1 plays important roles in producing inflammatory responses and modulating transcriptional networks during the development of colitis.

Indoleamine 2,3–dioxygenase 1 (IDO1, also known as tryptophan pyrrolase) is a heme-containing enzyme that catalyzes the oxidative cleavage of L-tryptophan to N-formyl-L-kynurenine in the first and rate-limiting step of tryptophan metabolism^1^. The great majority of tryptophan is known to be metabolized by the kynurenine pathway in which tryptophan is degraded through a series of metabolic reactions^2^,^3^. It has been reported that IDO1 is ubiquitously expressed in the colon^4^, small intestine^5^, brain^6^, spleen^7^, lung^8^ and epididymis^9^ as well as in myeloid cells including macrophages and dendritic cells^10^. Although the highest levels of expression were found in the colonic intestinal tissues^4^, the specific function of IDO1 in this location has not been fully studied.

IDO1 has attracted considerable attention in recent years because of its major roles in non-metabolic functions. The first discovery of the biologic relevance of IDO1 occurred in the placenta, where maternal T cells regulate immunity during gestation. These studies used 1-methyl-tryptophan (1-MT), an inhibitor of IDO1 activity^11^, and showed that IDO1 regulated T cell activity by suppressing proliferation. In addition, several other studies have shown an immunosuppressive role of IDO1 in conditions involving T cell activation such as graft-versus-host disease (GVHD)^12^,^13^, HIV infection^14^ and immunodeficiency diseases^15^. It has been shown that enhanced expression of IDO1 increased the production of tryptophan metabolites, which promotes cell-cycle arrest of and apoptosis of T cells and induce the differentiation of T regulatory cells (T_regs_)^16^.

Despite knowledge of the immune-modulatory role of IDO1, the mechanism by which IDO1 mediates inflammatory reactions is a field of active investigation and remains controversial. A contradictory effect of IDO1 on inflammatory responses during the development of colitis was recently described. Mechanistic studies using trinitrobenzene sulfonic acid (TNBS)-induced colitis suggested that inhibition of IDO1 leads to increased severity of colitis due to down-regulation of T_reg_ cell responses within the intestinal tract^17^,^18^. Conversely, another study reported that local increases in IDO1 production during active inflammatory responses resulted in more severe colitis promoted by key mediators of pro-inflammatory signaling^19^. Most previous results were drawn from studies of T cell-associated functions of IDO1. For example, TNBS is a chemical that induces colitis in a T cell-dependent manner^17^ as a result of delayed-type hypersensitivity reaction to haptenized proteins; however, because other studies raised the points that IDO1 may play inflammatory roles in a T cell-independent manner, different approaches are needed to elucidate the role of the enzyme in the development of colitis. One of the potential approaches is to study the roles of IDO1 using dextran sulfate sodium (DSS) which induces colitis by disrupting epithelial barrier function of colon tissues.

In this study, we performed for the first time an array-based transcriptome analysis to identify differentially-expressed genes targeted by IDO1 using *Ido1* knock-out (*Ido1*^−/−^) mice. We identified new molecular targets of IDO1 and described functionally distinct molecular mechanisms regulated by IDO1. In addition, we further examined the pathophysiological roles played by IDO1 in colitis development in studies using DSS-induced colitis model. We determined the effects of *Ido1* expression on colitis-related clinical parameters and histopathological damage. We also assessed changes in inflammatory cell recruitment using flow cytometry, and performed gene expression profiling analyses. To our knowledge, this is the first report of a comprehensive gene expression profile analysis of *Ido1*^−/−^ mice in either non- stimulated or DSS-stimulated conditions. This large-scale profiling may enhance our understandings of the contributions of IDO1 to colitis development, and provide novel target genes regulated by IDO1.

## Results

### Identification of differentially expressed genes in *Ido1* knock-out mice

**Figure 1:**
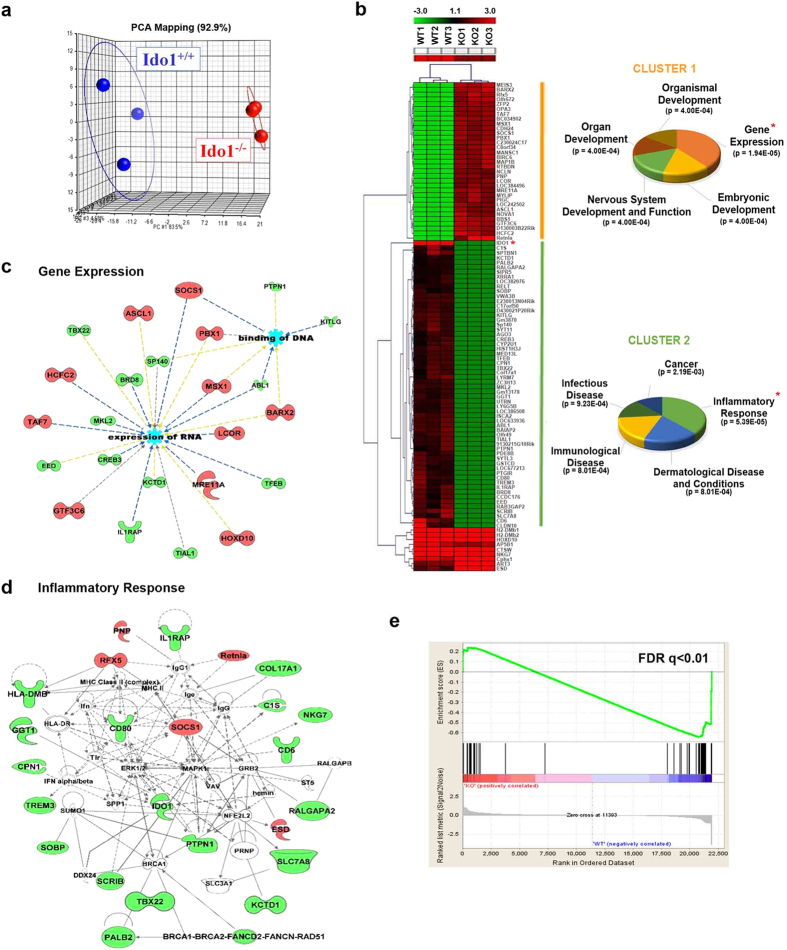
Differential gene-expression profiles of *Ido1*^−/−^ mice compared with *Ido1*^+/+^ mice detected by microarray analysis. (**a**) 3-D view of PCA scores plot of *Ido1*^−/−^ group (n = 3) *versus Ido1*^+/+^ group (n = 3). Groups are shown by different colors and dots represent individual strains. Blue spots represent *Ido1*^+/+^ group and the red spots represent *Ido1*^−/−^ group. On a 3D-PCA plot of *Ido1*^−/−^ group can be clearly distinguished from *Ido1*^+/+^ group. (**b**) Hierarchical clustering and heat map of up- or down-regulated genes that are differentially expressed (>2-fold, 1% [FDR]) in the absence of IDO1. Red indicates high relative expression and green indicates low expression of genes as shown in the scale bar. Cluster analysis functionally categorized by IPA; Canonical pathway significantly detected in *Ido1*^−/−^ mice compared to *Ido1*^+/+^ mice. Statistical significance of pathway modulation was calculated via a right-tailed Fisher’s exact test in Ingenutity Pathway. (**c**,**d**) Gene-pathway networks using IPA. (**c**) Gene Expression and (**d**) Inflammatory Response pathway are displayed as networks. Green and red symbols denote down-regulated and up-regulated genes in *Ido1*^−/−^ compared with *Ido1*^+/+^ mice, respectively. Arrows with unbroken lines indicate a direct interaction between two molecules, with the mode of action in the direction of the arrow; arrows with broken lines denote an indirect interaction. (**e**) We performed gene-set-enrichment analysis (GSEA) to determine whether the filtered gene list from *Ido1*^−/−^ mice *versus Ido1*^+/+^ mice showed specific enrichment in the inflammatory response in the rank-based analysis. Rank of 102 genes in our data sets ordered by expression level with enrichment plots for the up-regulated and down-regulated genes. ES (Enrichment Score) is a value that represents how well the gene set is enriched within the selected gene list. The FDR q value <0.01 for specific enrichment of the gene set is as indicated. The leading edge analysis of our microarray data identified that the gene is highly correlated with Inflammatory Response.

As a first attempt to identify the targets of IDO1, which catalyzes the first and rate-limiting step of tryptophan degradation, transcriptome analysis was performed using *Ido1*^−/−^ and *Ido1*^+/+^ mice. Total RNAs from the colon tissue of each mouse was respectively applied to Illumina Mouse WG-6 v.2 BeadChip arrays containing a total of 45,281 transcripts. Principal component analyses (PCA) confirmed that the gene expression profiles of *Ido1*^−/−^ mice were readily distinguishable from those of *Ido1*^+/+^ mice as shown in Fig. 1a. Differentially expressed genes in *Ido1*^−/−^ and *Ido1*^+/+^ mice were identified by unpaired t-test with thresholds of 1% false discovery rate [FDR] and 2-fold change restriction. A total of 102 significant genes were identified with 37 up- and 65 down-regulated genes. We confirmed that transcripts of *Ido1* (IDO1, marked by an asterisk in Fig. 1b) were completely absent in *Ido1*^−/−^ mice. To gain a broader understanding of the biological processes regulated by IDO1, we performed a series of bioinformatic analyses. Hierarchical clustering analysis showed three distinct gene clusters (Fig. 1b). Cluster 1, composed of up-regulated genes in *Ido1*^−/−^ mice was classified further to explore biological implications. Functional classification and Fisher’s exact test identified Gene Expression, Embryonic Development, Nervous System Development and Function, Organ Development, and Organismal Development as the top 5 significant biological processes. Accordingly, the expression of many genes in the gene expression-related transcriptional network was significantly increased including *Hoxd10*, *Barx2*, *Msx1*, and *Pbx1* (Fig. 1c). Likewise, the top five molecular and cellular functions of genes in cluster 2 were Inflammatory Response, Dermatological Disease and Conditions, Immunological Disease, Infectious Disease, and Cancer. The most significant category modulated by the absence of IDO1 was Inflammatory Response (P = 5.39E-05). These included *Cd6*, *Scrib*, *Il1rap*, *Cd80*, and *Trem3* in *Ido1*^−/−^ mice, suggesting that IDO1 might be an important mediator in inflammatory response (Fig. 1d). In addition, gene set enrichment analysis (GSEA) confirmed that genes associated with the inflammatory response were significantly enriched in the set of differentially expressed genes between *Ido1*^−/−^ and *Ido1*^+/+^ mice (FDR q < 0.01, Fig. 1e). These findings prompted us to explore the roles of IDO1 in colonic inflammatory responses.

### Contribution of IDO1 to development of DSS-induced colitis

**Figure 2:**
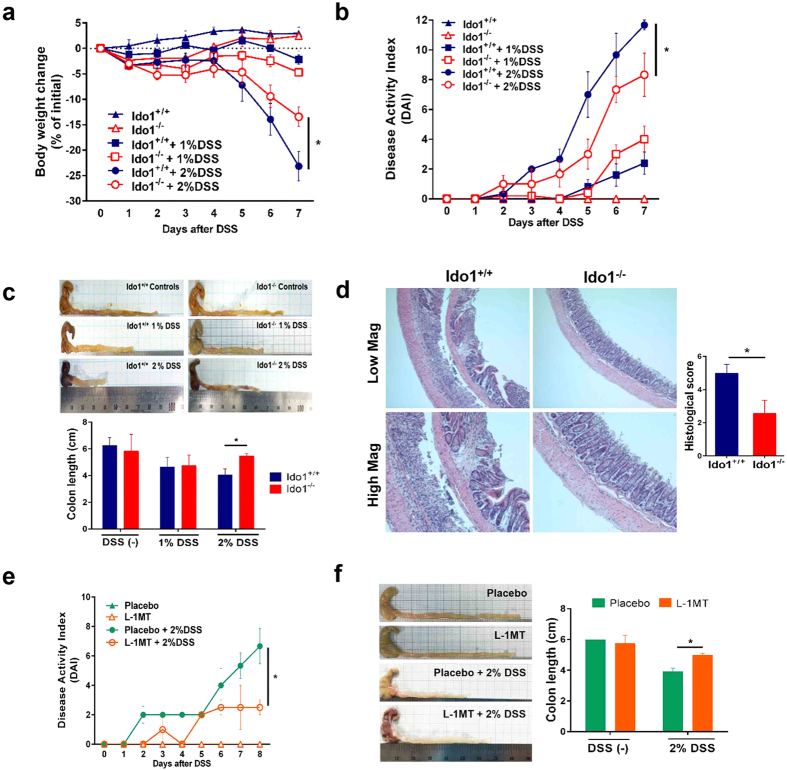
Phenotypic comparisons of DSS-induced colitis outcomes in gene deletion (*Ido1*^−/−^ *vs. Ido1*^+/+^ mice) and pharmacologic inhibition (placebo *vs.* L-1MT). (**a–c**) All data presented as the mean ± S.E.M of each genotype (n = 4–8 per group). Unpaired student’s t test was used to determine the significant difference between 2% DSS treatment gorups. *(asterisk) indicates the significant difference at p < 0.05. (**a**) Body weight curves of *Ido1*^−/−^ and *Ido1*^+/+^ mice in an acute model of DSS-induced colitis for 7 days. (**b**) Stool consistency, fecal bleeding and weight loss were observed on daily basis and DAI (Disease acitivity index) was scored for each mouse in *Ido1*^−/−^ and *Ido1*^+/+^. DAI score was graded on a scale of 0–4 as describe in the Methods. (**c**) Representative image of the DSS-induced colitis in *Ido1*^−/−^ and *Ido1*^+/+^ mice. Colon length of DSS-induced mice was measured on the 7^th^ day after the start of DSS treatment. (**d**) Representative H&E staining of colon tissue sections of each genotype treated with 1% DSS. Scale bars shows magnification 100 μm for upper pannels and 200 μm for lower pannels. Histological score is calculated by the sum of severity of inflammation (0–3), damage (0–4), and extension (0–4). Data are presented as mean ± S.E.M (n = 3–5 per group) and *p* value was estimated by unpaired t test. Bar with *indicates the significant difference at p < 0.05. (**e–f**) All data are presented as mean ± S.E.M of each group (n = 3 per group). Unpaired student’s t test was used to determine the significant difference between 2% DSS treatment gorups. *(asterisk) indicates the significant difference at p < 0.05. (**e**) Stool consistency, fecal bleeding and weight loss were observed on daily basis and DAI was scored for each mouse in placebo (*Ido1*^+/+^) and L-1MT (*Ido1*^+/+^ + L-1MT). (**f**) Representative image of the DSS-induced colitis in plcebo and L-1MT mice. Colon length of DSS-induced mice was measured on the 8^th^ day after the start of DSS treatment.

Based on the array analyses showing that the inflammatory response was the most significant biological process affected by the absence of IDO1 in untreated mice, we next sought to study the effects of IDO1 deficiency on colitis development. We used an established murine model of colitis induced by oral administration of DSS, a reagent that disrupts the barrier function of mucosal epithelial cells^20^. *Ido1*^−/−^ and *Ido1*^+/+^ mice were given either 1% or 2% DSS in their drinking water for 7 days and daily water intakes were measured. There was no difference in DSS-containing water consumption among all groups (see Supplementary Fig. S1). While treatment with 2% DSS resulted in a marked inflammatory reaction, 1% DSS treatment did not produce significant inflammatory reaction compared to the 0% control in *Ido1*^+/+^ mice. In treatment with 2% DSS, progressive weight loss, increased disease activity index (DAI) including severe diarrhea and intestinal bleeding, and greater shortening of colon length were detected in *Ido1*^+/+^ compared to *Ido1*^−/−^ mice (Fig. 2a–c). These results suggest that IDO1 may play roles in promoting the inflammatory response in DSS-challenged mice. In addition, although treatment with 1% DSS did not induce significant differences in body weight and DAIs between *Ido1*^−/−^ and *Ido1*^+/+^ mice, it caused histological changes showing that the extent of tissue damage in *Ido1*^+/+^ mice was much greater than those for *Ido1*^−/−^ mice (Fig. 2d). Inflammatory cell infiltration and crypt damage was more apparent in these mice as evidenced on H&E staining.

Furthermore, to explore whether pharmacological inhibition of IDO1 reproduces the results observed following *Ido1* gene deletion, we administered *Ido1*^+/+^ mice with L-1MT, a specific IDO1 inhibitor, then followed by colitis induction with 2% DSS treatment. IDO1 blockade by L-1MT ameliorated severe diarrhea and intestinal bleeding, resulting in significant reduction in the DAI. Shortening of colon length was notably attenuated in mice administered with L-1MT compared to the placebo (Fig. 2e,f). Taken together, our data indicate that the severity of DSS-induced colitis development was significantly reduced in *Ido1*^−/−^ mice and L-1MT administered mice, suggesting that IDO1 deficiency might protect against pro-inflammatory signals. We next carried out transcriptome analysis to elucidate the underlying molecular mechanisms of this effect.

### Gene expression profiling analysis of inflamed colon tissues in *Ido1* ^−/−^ mice

**Figure 3:**
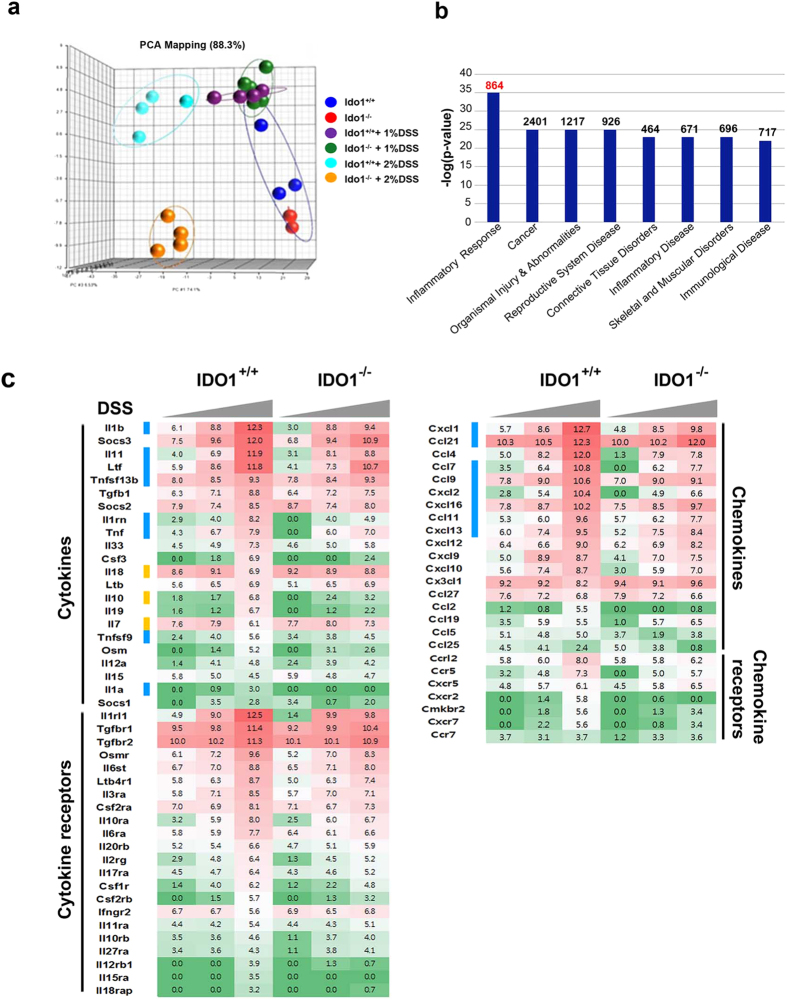
Transcriptional Profiling of inflamed colon tissues in *Ido1*^+/+^ and *Ido1*^−/−^ mice after DSS treatment. (**a**) 3-D view of PCA scores plot of data obtained on *Ido1*^−/−^ mice *versus Ido1*^+/+^ mice treated with DSS. On a 3D-PCA plot of 2% DSS treated *Ido1*^−/−^ group can be significantly seperated from 2% DSS treated *Ido1*^+/+^ group. Each spots represents individual mouse in the group. Blue spots, *Ido1*^+/+^ group; Red spots, *Ido1*^−/−^ group; Purple spots, *Ido1*^+/+^ + 1% DSS treatment group, Green spots, *Ido1*^−/−^ + 1% DSS treatment group; Sky blue spots, *Ido1*^+/+^ + 2% DSS treatment group; Orange spots. *Ido1*^−/−^ + 2% DSS treatment group. (**b**) Functional categories by IPA; Canonical pathway significantly detected in *Ido1*^−/−^ mice compared to *Ido1*^+/+^ mice after DSS-induced colitis. Statistical significance of pathway modulation was calculated via a right-tailed Fisher’s exact test in Ingenutity Pathway and represented as –log (p-value). A larger value on the x axis indicates a higher degree of significance, i.e., a smaller p value. The leading edge analysis of our microarray data identified gene highly correlated with Inflammatory Response. (**c**) Heat map of cytokine and chemokine genes that are differentially expressed [5% FDR and 2-fold change restriction] in DSS-challenged *Ido1*^−/−^ *versus Ido1*^+/+^. The given values are the average of normalized intensities (3–5 mice per group) with red representing higher levels of expression and green indicating lower levels. Color code: blue, pro-inflammatory cytokines; yellow, anti-inflammatory cytokines.

**Figure 4:**
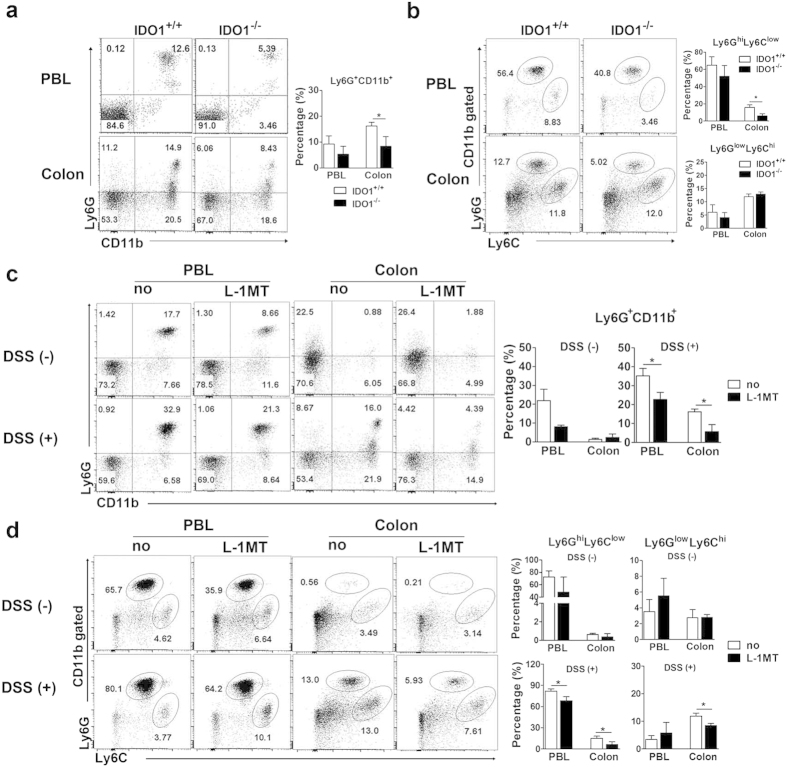
Flow cytometric analysis of the CD11b^+^Gr-1^+^ cell responses in the presence or absence of IDO1 in DSS-induced colitis. (**a**) The expression level of CD11b^+^Gr-1^+^ and (**b**) the proportion of Ly6G^hi^Ly6C^low^ and Ly6G^low^Ly6C^hi^ populations in the gated CD11b^+^ cells were analyzed by flow cytometry. PBLs and colon cells from 2% DSS treated *Ido1*^+/+^ and *Ido1*^−/−^ mice and non-treated control mice on days 7 and 8 were stained with FITC-conjugated or eFluor® 450-conjugated anti-Ly6G, PE-conjugated with anti-CD11b and APC-conjugated anti-Ly6C Abs. (**c**) The effects of L-1MT on the expression of CD11b^+^Gr-1^+^ cell in DSS-induced colitis*. Ido1*^+/+^ mice were orally administered with L-1MT at 400mg/kg per dose or control vehicle twice daily. On day 5 of the experiment, mice were received 2% DSS in drinking waters and sacrificed on day 13. PBLs and colon cells were harvested from and analyzed the expression of CD11b^+^Gr-1^+^ and (**D**) the proportion of Ly6G^hi^Ly6C^low^ and Ly6G^low^Ly6C^hi^ populations in the gated CD11b^+^ cells were analyzed by flow cytometry. Representative flow cytometry data are shown. *p < 0.05.

We analyzed the gene expression profiles of three to five *Ido1*^−/−^ or *Ido1*^+/+^ mice from the controls and from cohorts treated with 1% or 2% DSS (Fig. 3a). As expected, the transcription levels of *Ido1* were significantly increased in the inflamed colon tissues of *Ido1*^+/+^ mice, suggesting that the inflammatory response induced *Ido1* transcription (data not shown). PCA analysis showed that *Ido1*^−/−^ mice had a distinct pattern compared with *Ido1*^+/+^ mice only in the 2% DSS treatment group (Fig. 3a). We performed a one-way ANOVA analysis to identify differentially-expressed genes with statistical thresholds of 5% FDR and 2-fold change restriction among the six groups. A total of 6,421 genes were identified as significant and were classified further based on their biological functions. As expected, the Inflammatory Response category was the most significant key function (p = 1.23E-35 to 3.89E-08) (Fig. 3b). In comparisons of *Ido1*^−/−^ and *Ido1*^+/+^ mice, much higher numbers of genes were differentially expressed under DSS-stimulated conditions than in the basal non-stimulated state. Cytokines including interleukins and interferons are known to induce a broad inflammatory reaction in response to infection or injury^21^,^22^,^23^. This prompted us to further investigate cytokine and chemokine gene expression signatures. Strikingly, the expression levels of pro-inflammatory cytokines and chemokines, such as *Il-1β*, *TNF, Cxcl1,* and *Ccl7*, which are central mediators of inflammation were significantly elevated in a dose-dependent manner by DSS treatment in *Ido1*^+/+^ mice, however, the elevation was substantially attenuated in *Ido1*^−/−^ mice (Fig. 3c). In contrast, IL-7 and IL-18 are anti-inflammatory cytokines and their expression was much lower in DSS-treated *Ido1*^+/+^ compared with DSS-treated *Ido1*^−/−^ mice. These gene expression signatures suggested that *Ido1*^−/−^ mice would be less likely to develop severe colitis than *Ido1*^+/+^ mice. The pro-inflammatory cytokines and chemokines noted above were previously reported to be induced in inflammatory cells, including monocytes, as part of the inflammatory reaction^24^,^25^. These findings led us to assess CD11b^+^Gr-1^+^ cells in peripheral blood (PBL) and colon using FACS. The frequencies of CD11b^+^Gr-1^+^ (Ly6G) cells were higher in the PBL and colon of *Ido1*^+/+^ mice than in *Ido1*^−/−^ mice following treatment with 2% DSS, although the difference in PBL was not significant (Fig. 4a). Interestingly, in the CD11b^+^Gr-1^+^ cells, the difference in the proportion of the Ly6G^hi^Ly6C^low^ subpopulation was significant between the two genotypes of mice with 2% DSS-treatment: the proportion of Ly6G^hi^Ly6C^low^ was higher in the *Ido1*^+/+^ mice than in the *Ido1*^−/−^ mice (Fig. 4b). To verify the role of IDO1 in the expansion of CD11^+^Gr-1^+^ cells in the DSS-induced colitis model, we examined whether treatment with the IDO1 inhibitor, L-1MT, would reproduce the results from *Ido1*^−/−^ mice. Consistently, the frequency of CD11^+^Gr-1^+^ cells and the proportion of the Ly6G^hi^Ly6C^low^ subpopulation in CD11^+^Gr-1^+^ cells were lower in the L-1MT-treated mice with DSS-induced colitis, compared with their vehicle-treated control counterparts (Fig. 4c,d). Moreover, the proportion of colonic granulocytes (CD11b^+^Ly6G^+^Ly6C^low^F4/80^-^CD11c^−^)^26^ was significantly lower in both *Ido1*^−/−^ and L-1MT-treated *Ido1*^+/+^ mice compared to the *Ido1*^+/+^ control mice after DSS treatment (see Supplementary Fig. S2a). However, no significant difference was observed in monocyte populations (CD11b^+^Ly6C^+^Ly6G^-^CD11c^−^)^26^ among the three groups (Supplementary Fig. S2b). This indicates that loss of the functional activity of IDO1 may contribute to reduced expansion of CD11b^+^Gr-1^+^ cells including granulocytes in the DSS-induced colitis model. These results suggest that IDO1 induced a systemic inflammatory response including a gut inflammatory response associated with increased expression of pro-inflammatory cytokines and chemokines.

### IDO1 deficiency results in down regulation of TLR and NF-kB signaling pathways

**Figure 5:**
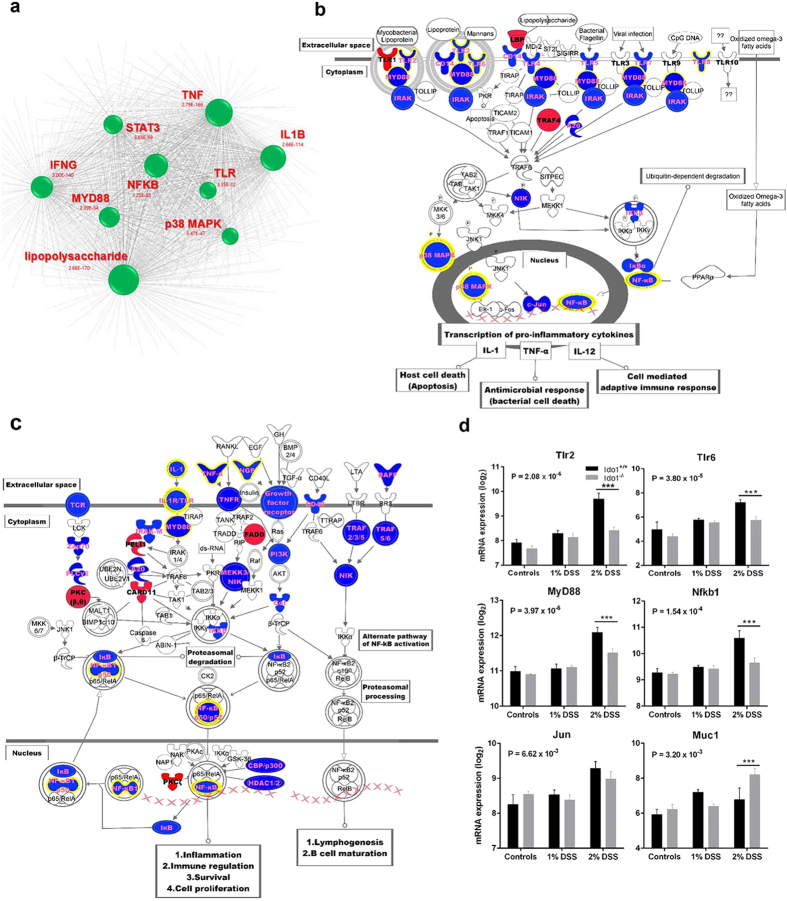
Down-regulation of TLR and NF-kB signaling pathway in *Ido1*^−/−^ mice. (**a**) Upstream regulator analysis of the genes observed expression changes using network visualization. Groups identified are shown in circles. Size of the nodes is inversely proportional to the z-score. (**b**) Pathway model of TLR signaling based IPA knowledge. Genes in blue color indicate down-regulation and in red color indicate up-regulation in DSS-challenged *Ido1*^−/−^ mice *versus Ido1*^+/+^ mice. (**c**) Pathway model of NF-kB signaling based IPA knowledge. Genes in blue color indicate down-regulation and in red color indicate up-regulation in DSS-challenged *Ido1*^−/−^ mice *versus Ido1*^+/+^ mice. (**d**) Differential expression of a selection of genes associated with TLR signaling (*Tlr2*, *Tlr6*, and *MyD88*), NF-kB signaling (*Nfkb1* and *Jun*), and colonic transcript level of *Muc1* gene in DSS-challenged *Ido1*^−/−^ *versus Ido1*^+/+^ mice. Data are presented as means ± S.E.M (n = 3–5 for each group). One-way ANOVA was used to determine the significant difference among the groups and unpaired student’s t test was used to determine the significant difference between 2% DSS treatment gorups. *p < 0.05 and ***p < 0.01.

We next conducted an upstream regulator analysis to identify major upstream molecules of the differentially expressed genes identified in our data set^27^. It would be critical to identify the upstream regulatory molecules and their associated mechanisms of action to provide biological insights into the observed expression changes. In addition to transcription factors, upstream regulators can include any gene or small molecule that has been observed experimentally to affect gene expression directly or indirectly. The top 9 predicted upstream regulators ranked by z-score are lipopolysaccharide (p = 2.66E-170), TNF (p = 2.79E-166), IFNG (p = 3.00E-140), IL1B (p = 2.66E-114), NF-kB complex (p = 1.25E-83), STAT3 (p = 3.65E-69), MYD88 (p = 2.39E-54), p38 MAPK (p = 5.47E-47), and TLR (p = 1.51E-45) (Fig. 5a). Surprisingly, all regulators are well known contributors to the development of inflammatory responses. As a point of interest, the greatest overlap with the regulators was in the pathways related to the Toll-like receptor and NF-kB signaling. Given these findings, we focused on genes belonging to those pathways in more detail. The most striking result was that expression of the majority of genes involved in TLR signaling was down-regulated in DSS-treated *Ido1*^−/−^ mice, suggesting that TLR signaling is essential for the contributions of IDO1 to the development of DSS-induced colitis (Fig. 5b). In the basal state (non-stimulated), expressions of *Tlr2*, *Tlr6*, and *Myd88* remained low and the levels were similar in *Ido1*^−/−^ and *Ido1*^+/+^ mice. However, treatment with DSS resulted in dramatic increases in the expressions of those genes in *Ido1*^+/+^ mice in a dose-dependent manner. The expressions of these genes were not significantly induced by DSS treatment in IDO1-deficient mice (Fig. 5d). Importantly, MyD88 is a key adaptor protein in the signal transduction cascades shared by most TLRs and MyD88-dependent TLR signaling pathways were found to be down-regulated in our study (Fig. 5b). This suggests that the differences in colitis development between *Ido1*^−/−^ and *Ido1*^+/+^ mice result from dysregulation of the TLR-MyD88 signaling pathway. We also ascertained that TLR-triggered cascades downstream of NF-kB signaling molecules like NF-kB (nuclear factor kB, p = 1.54E-4), JNKs (JUN N-terminal kinases, p = 6.62E-3), and IRF5 (interferon regulatory factor 5, p = 1.20E-3), which were all down-regulated in DSS-treated *Ido1*^−/−^ mice (Fig. 5c). These results implied that in mice with DSS-induced colitis, TLR signaling was inhibited by IDO1-deficiency, which suppressed pro-inflammatory cytokine and chemokine production through the regulation of a multitude of transcription factors such as NF-kB.

## Discussion

IDO1-mediated tryptophan metabolism in various tissues is linked to numerous biological and physiological functions. Heightened expression of IDO1 in colonic intestinal tissues led to the hypothesis that IDO1 plays critical roles in gut homeostasis. Various microorganisms and food antigens exist in the lumen and challenge the intestinal immune system. As the luminal surface of the gastrointestinal tract continually interacts with foreign antigens such as pathogenic bacteria, the mucosal immune system maintains immune tolerance to limit inflammatory responses elicited by these antigens. In the present study, we showed that in the basal state in which there was no chemical induction of inflammation, IDO1 deficiency did not lead to pathophysiologic changes in the gut. However, gene expression profiling analysis revealed that a transcriptional network of inflammatory responses was significantly down-regulate in the absence of IDO1.

When the mucosal immune system fails to maintain tolerance, pathogenic microbes are able to infect the host. Impaired immune tolerance in the intestine can lead to inflammatory bowel diseases such as Crohn’s disease and ulcerative colitis^28^,^29^,^30^. Treatment with DSS treatment mimics the condition of impaired immune tolerance because it disrupts the mucosal barrier of epithelial cells on the luminal surface. Studies of the DSS-induced colitis model system enabled us to characterize the role of IDO1 in driving inflammatory response, which is not apparent in the non-stimulated state. We showed that DSS-treated IDO1-deficient mice did not develop colitis of the same severity as normal control mice by assessing the loss in body weight, intestinal bleeding, diarrhea, shortening of colon length and histological lesions. It should be noted that there are conflicting results regarding the role of IDO1 in different mouse models of colitis. In studies that utilized TNBS to induce colitis, inhibition of IDO1 was found to exacerbate colitis^17^,^31^. This discrepancy can probably be explained, at least in part, by the fact that TNBS induces a T cell-dependent disease while the DSS induction is in a different manner. Although both DSS and TNBS colitis are used for inflammatory bowel disease animal model, DSS colitis has been distinguished from TNBS models by many others in the several points^20^,^32^. For instance, while TNBS-induced colitis develops as a result of delayed-type hypersensitivity reaction to haptenized proteins, DSS-induced colitis is the result of a change in epithelial barrier function^32^. Additionally, it is known that while TNBS induces Crohn’s disease-like colitis, DSS induces ulcerative colitis^20^. Therefore, disease exacerbation in IDO1-deficient TNBS-treated mice was attributed to the inhibition of T_reg_ activity^17^,^31^, which is a distinct mechanism from what we found in IDO1-deficient DSS-treated mice.

It is well established that TLRs and NF-kB signaling pathways make important contributions to inflammatory responses. Strikingly, our transcriptome analyses showed that the expression of members of the TLR-MyD88-NF-kB signaling pathways was significantly decreased in the absence of IDO1. Consequently, down-regulation of those signaling pathways resulted in dramatic changes in the expression of cytokines and chemokines. We showed that in IDO1 deficient mice, the frequencies of circulating inflammatory cells in peripheral blood and in the gut were decreased as well. These findings suggest that IDO1 may require TLR-MyD88-NF-kB signaling to promote the development of colitis. It is notable that compared to normal mice, IDO1-deficient mice had higher expression of *Muc1,* which encodes mucin protein, the first line of host defense against invading bacteria (Fig. 5d, p = 3.20E-3). Reduction in the expression of *Muc1* severely affects epithelial barrier function^33^,^34^. Several studies reported that mucin may be a negative regulator of TLR signaling^35^,^36^. This suggests that the absence of IDO1 may lead to increased mucin expression and subsequently down-regulate TLR-MyD88-NF-kB signaling.

IDO1 has become an emerging target for the treatment of cancer, infection, autoimmunity, and other diseases associated with inflammatory responses and immunosuppression^37^,^38^,^39^,^40^. The present report utilizing the *Ido1*^−/−^ mouse model provides the first comprehensive analysis of IDO1 targets at the transcriptome level and broadens our understanding of the diverse functions of IDO1 during inflammatory responses. This study also suggest that IDO1 is likely to be a promising target of therapeutic intervention in colitic diseases.

## Methods

### Mice

*Ido1*^−/−^ mice of the C57BL/6 (B6) genetic background were obtained from The Jackson Laboratory (Bar Harbor, ME, USA). *Ido1*^−/−^ mice were crossed with C57BL/6 wild-type (The Jackson Laboratory) to generate the *Ido1*^−/−^ and *Ido1*^+/+^ offspring used in this study. Genotypes of knockout mice were verified via PCR typing. The mice used were 10–12 weeks old and weighed 18–23 g. Age- and weight-matched female littermates were used as controls. C57BL/6 mice and *Ido1*^−/−^ mice were maintained under specific pathogen free (SPF) condition at the Center of Animal Resource Development, Seoul National University College of Medicine. The mice were maintained based on the guidelines of Seoul National University Animal Experiment Ethics Committee. All animal experimental protocols were approved by the Committee on the Ethics of animal experiments of Seoul National University (Institutional Animal Care and Use Committee permit number: SNU-150119-5). All experiments were carried out in accordance with the guidelines and regulations.

### Induction of colitis and evaluation of colitis severity

To generate an acute colitis experimental model, dextran sulfate sodium (DSS) (molecular mass 36–50 kDa; MP Biomedicals, Illkirch, France) was added to the drinking water at concentrations of 1% or 2% (w/v) given *ad libitum* for 7 days. Control mice received drinking water without DSS. The subsequent course of colitis development was evaluated by monitoring daily weight changes. Colitis severity also was scored by evaluating clinical disease activity through daily observation of the following parameters: weight loss (0 points = No weight loss or weight gain, 1 points = 5–10% weight loss, 2 points = 11–15% weight loss, 3 points = 16–20% weight loss, 4 points = >21% weight loss); stool consistency (0 points = normal and well formed, 2 points = very soft and unformed, 4 points = watery stool); and bleeding stool score (0 points = normal color stool, 2 points = reddish color stool, 4 points = bloody stool). The disease activity index (DAI) was calculated based on the combined scores of weight loss, stool consistency, and bleeding ranging from 0 to 12. All parameters were scored from day 0 to day 7. At the 7^th^ day after DSS-colitis induction, mice were sacrificed and the entire colon was quickly removed. After colon length was determined as a marker of inflammation, the entire colon was cut open lengthwise and gently flushed with sterile phosphate-buffered saline (PBS) to remove any traces of feces. Colon segments were immediately frozen in liquid nitrogen and stored at −80 °C for subsequent extraction of total RNA. For histological analysis, colon segments were fixed in 10% neutral buffered formalin phosphate and stored at room temperature until study for evidence of inflammation.

### Administration of L-1MT

9–11 weeks old *Ido1*^+/+^ mice were administered L-1MT. To prepare L-1MT for oral gavage, 1g of L-1MT (purchased from Sigma-Aldrich) was added to a 15 ml conical tube with 10 ml Methocel/Tween [0.5% Tween 80/0.5% Methylcellulose (v/v in water; both from Sigma-Aldrich)]. The mixture was bead milled overnight by adding 2–3 mm glass beads and mixing inversion. The next day, the L-1MT concentration was adjusted to 80 mg/ml by adding an additional 2.5 ml Methocel/Tween and mixing again. The L-1MT slurry was administered by oral gavage at 400 mg/kg/dose (100 μl of total volume) using a curved feeding needle (20-gquge 1^1/2^ in; Fisher) as previously described^41^. For twice a day dosing, L-1MT was administered once in the morning and once in the evening. On day 5 of the experiment, all mice received 2% DSS treatment and mice were sacrificed on day 13.

### Histological analysis of colitis

Routinely processed, 4–6 μm paraffin-embedded sections of colon samples were prepared and stained with hematoxylin and eosin (H&E) for histological grading. Histological scores, including severity of colitis, were evaluated in a blinded manner as previously described by Laroui *et al.*^42^. Grades were evaluated from 0–4 for the following three criteria: severity of inflammation (0, rare inflammatory cell in the lamina propria; 1, increased inflammatory cells in the lamina propria; 2, confluent inflammatory cells extending into the submucosa; and 3, transmural extension of the inflammatory cell infiltrate); damage (0, none; 1, loss of the basal 1/3 of the crypt; 2, loss of the basal 2/3 of the crypt; 3, loss of the entire crypt but intact epithelial cells; and 4, loss of the entire crypt and of the surface epithelial cells); extension (0, none; 1, focal; 2, lesion involving 1/3 of the intestine; 3, lesion involving 2/3 of the intestine; and 4, lesion involving the entire intestine). Scores for each criterion were added to give an overall inflammation score for each sample with a range of 0–11. The histological grades were determined for each section, and the sum of the grades was reported as the histological score for each mouse. The level of colitis was blindly assessed by two histopathologists.

### Flow cytometry (FACS) analyses

Fresh peripheral blood lymphocytes (PBLs) were prepared by incubating blood with ACK (ammonium-chloride-potassium) buffer to lyse red blood cells at room temperature for 3–5 minutes, and stained using FACS buffer (1X phosphate-buffered saline [PBS] with 0.1% bovine calf serum and 0.05% sodium azide). Colonic cells in the lamina propria of mice were isolated according to the previously described protocol^43^. Briefly, colon pieces (1 cm pieces) were treated with 5 ml of predigestion solution (1× HBSS containing 2 mM EDTA and 1 mM DTT) for 20 min at 37 °C. After incubation, cells epithelial cells were decanted and again incubated the pieces for 20 min at 37 °C. Intestinal pieces were washed with 1× PBS to remove remaining EDTA. And collected tissues were incubated with digestion solution (1.5 mg/ml of collagenase D [Roche], 0.1 mg/ml of DNase I [Sigma-Aldrich] and 5% of fetal bovine serum in 100 ml of 1× PBS) for 20 min at 37 °C and repeated. Cells were harvested and centrifuge for 10 min at 1,500 rpm, and resuspended with 5 ml of 40% percoll solution and overlayed to 80% percoll solution. Cells were centrifuged for 20 min at 1,000g and resuspended with FACS buffer. And cells were stained FITC- and eFluor® 450-conjugated anti-Ly6G (Gr-1; RB6-8C5, eBioscience, San Diego, CA, USA), PE- and PE-Cy7-conjugated anti-CD11b (M1/70, eBioscience), APC-conjugated anti-Ly6C (HK1.4, eBioscience), PE-Cy5-conjugated anti-F4/80 (BM8, eBioscince), APC-Cy7-conjugated anti-CD11c (N418, Biolegend, San Diego, CA, USA) antibodies at 4 °C for 30 min. After washing with FACS buffer, the cells were analyzed by a FACS Calibur (BD Bioscience, Franklin Lakes, NJ, USA) and FACS LSRII (BD Bioscience) and Flowjo software (Tree star, Ashland, OR, USA).

### Microarray hybridization

The gene expression profile was determined using the MouseWG-6 v.2 Expression BeadChips (Illumina®). For microarray hybridization, total RNA was isolated by homogenizing colon tissue samples and was purified using a DNA-free RNA isolation kit (RNAqueous-4PCR kit; Ambion, Austin, TX, USA) in accordance with the manufacturer’s instructions. Total RNA integrity and quantity were assessed with a Nanodrop-2000 Spectrophotometer (Thermo Fisher Scientific, Wilmington, DE). Only total RNA with an OD 260/280 ratio >2.0 was used for microarray hybridization. RNA samples were first amplified for array analyses using the Illumina Total Prep RNA Amplification Kit (Ambion, Austin, TX, USA) according to the manufacturer’s instructions. Briefly, 500ng of total RNA, isolated from colon tissue, was used to prepare labelled cRNA with overnight incubation according to the manufacturer’s protocol. The quality and quantity of the labelled cRNA were monitored using a Nanodrop-2000 Spectrophotometer. Amplified cRNA (1.5 μg) was hybridized on MouseWG-6 Expression BeadChip arrays, containing more than 45,281 well-annotated Ref transcripts, according to the manufacturer’s standard protocol. The arrays were then scanned on a BeadArray Reader (BeadStation 500G Instrument, Illumina Inc.), and Spot images identification and quantification were obtained by the Genome Studio software v1.0.2. (Illumina Inc.).

### Identification of significant genes

The raw data were pre-processed through three steps: background correction was performed, the data were then log-transformed to log 2 scale, and normalized by quantile normalization method implemented in the Genome Studio software (Illumina Inc.). Significant difference between two genotypes in each dose (0% DSS [baseline], 1% DSS treatment, 2% DSS treatment), differences between dose response effect in each genotype, and difference between genotype x dose interaction were identified using ANOVA test (*p* < 0.05) on log 2-transformed normalized intensities using by Partek® Genomics Suite software v6.3 (Partek, St Louis, MI) (http://www.partek.com/partekgs). Transcripts with more than 2-fold differential expression and a false discovery rate (FDR) < 0.01 were selected for each specific comparison analyzed.

### Functional enrichment and clustering analysis

Functional categorization analysis was performed based upon gene ontology consortium (GO). Broad Gene Set Enrichment Analysis (GSEA) was done to examine the significance of each functional category classified by GO^44^ (http://www.broadinstitute.org/gsea/index.jsp). Hierarchical clustering analysis was carried out with Genesis software v1.7.5^45^ using the Pearson correlation distance matrix with average linkage algorithm.

### Statistical Analysis

Data were expressed as the mean ±S.E.M. Statistical significance (*p* value < 0.05) was evaluated either by unpaired student’s *t* test between two groups or one-way analysis of variance (ANOVA) to compare multiple groups using dose and genotype as factors. Statistical analyses were performed using Graph Pad Prism 5 software (GraphPad Software Inc., La Jolla, CA, USA) and SAS Enterprise Guide 6.1 (SAS Institute Inc., Cary, NC, USA). For gene analyses, significances for functional enrichment of specific genes were determined by a right-tailed Fisher’s exact test as the negative log of the probability that the number of focus genes is not due to random chance.

## Additional Information

**How to cite this article**: Shon, W.-J. *et al.* Severity of DSS-induced colitis is reduced in Ido1-deficient mice with down-regulation of TLR-MyD88-NF-kB transcriptional networks. *Sci. Rep.* **5**, 17305; doi: 10.1038/srep17305 (2015).

## Supplementary Material

Supplementary Information

## References

[b1] Takikawa O., Yoshida R., Kido R. & Hayaishi O. Tryptophan degradation in mice initiated by indoleamine 2, 3-dioxygenase. J Biol Chem. 261, 3648–3653 (1986). 2419335

[b2] Rios-Avila L., Nijhout H. F., Reed M. C., Sitren H. S. & Gregory J. F. A mathematical model of tryptophan metabolism via the kynurenine pathway provides insights into the effects of vitamin B-6 deficiencky, tryptophan loading, and induction of tryptophan 2, 3-dioxygenase on tryptophan metabolites. J Nutr. 143, 1509–1519 (2013). 23902960 10.3945/jn.113.174599 PMC3743279

[b3] Stavrum A.-K., Heiland I., Schuster S., Puntervoll P. & Ziegler M. Model of tryptophan metabolism, readily scalable using tissue-specific gene expression data. J Biol Chem. 288, 34555–34566 (2013). 24129579 10.1074/jbc.M113.474908 PMC3843069

[b4] Matteoli G. *et al.* Gut CD103+ dendritic cells express indoleamine 2, 3-dioxygenase which influences T regulatory/T effector cell balance and oral tolerance induction. Gut 59, 595–604 (2010). 20427394 10.1136/gut.2009.185108

[b5] Yoshida R. *et al.* Regulation of indoleamine 2, 3-dioxygenase activity in the small intestine and the epididymis of mice. Arch Biochem Biophys. 203, 343–351 (1980). 6967714 10.1016/0003-9861(80)90185-x

[b6] Kwidzinski E. *et al.* Indolamine 2, 3-dioxygenase is expressed in the CNS and down-regulates autoimmune inflammation. FASEB J. 19, 1347–1349 (2005). 15939737 10.1096/fj.04-3228fje

[b7] Boasso A. *et al.* Regulatory T-cell markers, indoleamine 2, 3-dioxygenase, and virus levels in spleen and gut during progressive simian immunodeficiency virus infection. J Virol. 81, 11593–11603 (2007). 17715231 10.1128/JVI.00760-07 PMC2168803

[b8] Romani L. *et al.* Defective tryptophan catabolism underlies inflammation in mouse chronic granulomatous disease. Nature 451, 211–215 (2008). 18185592 10.1038/nature06471

[b9] Jrad-Lamine A. *et al.* Indoleamine 2, 3-dioxygenase 1 (IDO1) is involved in the control of mouse caput epididymis immune environment. PLoS One. 8, e66494 (2013). 23840489 10.1371/journal.pone.0066494 PMC3688773

[b10] Mellor A. L. & Munn D. H. IDO expression by dendritic cells: tolerance and tryptophan catabolism. Nat Rev Immunol. 4, 762–774 (2004). 15459668 10.1038/nri1457

[b11] Munn D. H. *et al.* Prevention of allogeneic fetal rejection by tryptophan catabolism. Science 281, 1191–1193 (1998). 9712583 10.1126/science.281.5380.1191

[b12] Jasperson L. K. *et al.* Indoleamine 2, 3-dioxygenase is a critical regulator of acute graft-versus-host disease lethality. Blood 111, 3257–3265 (2008). 18077788 10.1182/blood-2007-06-096081 PMC2265461

[b13] Lu Y. *et al.* IFN-γ and indoleamine 2, 3-dioxygenase signaling between donor dendritic cells and T cells regulates graft versus host and graft versus leukemia activity. Blood 119, 1075–1085 (2012). 22130799 10.1182/blood-2010-12-322891 PMC3271719

[b14] Favre D. *et al.* Tryptophan catabolism by indoleamine 2, 3-dioxygenase 1 alters the balance of TH17 to regulatory T cells in HIV disease. Sci Transl Med. 2, 32ra36 (2010). 10.1126/scitranslmed.3000632 PMC3034445 20484731

[b15] Liu X. *et al.* Selective inhibition of IDO1 effectively regulates mediators of antitumor immunity. Blood 115, 3520–3530 (2010). 20197554 10.1182/blood-2009-09-246124

[b16] Terness P. *et al.* Inhibition of Allogeneic T Cell Proliferation by Indoleamine 2,3-Dioxygenase–expressing Dendritic Cells Mediation of Suppression by Tryptophan Metabolites. J Exp Med. 196, 447–457 (2002). 12186837 10.1084/jem.20020052 PMC2196057

[b17] Takamatsu M. *et al.* IDO1 Plays an Immunosuppressive Role in 2,4,6-Trinitrobenzene Sulfate–Induced Colitis in Mice. J Immunol. 191, 3057–3064 (2013). 23956437 10.4049/jimmunol.1203306

[b18] Gurtner G. J., Newberry R. D., Schloemann S. R., McDonald K. G. & Stenson W. F. Inhibition of indoleamine 2,3-dioxygenase augments trinitrobenzene sulfonic acid colitis in mice. Gastroenterology 125, 1762–1773 (2003). 14724829 10.1053/j.gastro.2003.08.031

[b19] Thaker A. I. *et al.* IDO1 metabolites activate β-catenin signaling to promote cancer cell proliferation and colon tumorigenesis in mice. Gastroenterology 145, 416–425 (2013). 23669411 10.1053/j.gastro.2013.05.002 PMC3722304

[b20] Strober W., Fuss I. J. & Blumberg R. S. The immunology of mucosal models of inflammation. Annu Rev Immunol. 20, 495–549 (2002). 11861611 10.1146/annurev.immunol.20.100301.064816

[b21] Henao-Mejia J., Elinav E., Strowig T. & Flavell R. A. Inflammasomes: far beyond inflammation. Nat Immunol. 13, 321–324 (2012). 22430784 10.1038/ni.2257

[b22] Teijaro J. R. *et al.* Persistent LCMV infection is controlled by blockade of type I interferon signaling. Science 340, 207–211 (2013). 23580529 10.1126/science.1235214 PMC3640797

[b23] Deretic V., Saitoh T. & Akira S. Autophagy in infection, inflammation and immunity. Nat Rev Immunol. 13, 722–737 (2013). 24064518 10.1038/nri3532 PMC5340150

[b24] Jiménez V. C. *et al.* Differential expression of HIV-1 interfering factors in monocyte-derived macrophages stimulated with polarizing cytokines or interferons. Sci Rep. 2, doi: 10.1038/srep00763 (2012). PMC3478582 23094138

[b25] Jagannathan-Bogdan M. *et al.* Elevated proinflammatory cytokine production by a skewed T cell compartment requires monocytes and promotes inflammation in type 2 diabetes. J Immunol. 186, 1162–1172 (2011). 21169542 10.4049/jimmunol.1002615 PMC3089774

[b26] Gabrilovich D. I., Ostrand-Rosenberg S. & Bronte V. Coordinated regulation of myeloid cells by tumours. Nat Rev Immunol. 12, 253–268 (2012). 22437938 10.1038/nri3175 PMC3587148

[b27] Krämer A., Green J., Pollard J. & Tugendreich S. Causal Analysis Approaches in Ingenuity Pathway Analysis (IPA). Bioinformatics 30, 523–530 (2014). 24336805 10.1093/bioinformatics/btt703 PMC3928520

[b28] Jostins L. *et al.* Host-microbe interactions have shaped the genetic architecture of inflammatory bowel disease. Nature 491, 119–124 (2012). 23128233 10.1038/nature11582 PMC3491803

[b29] Maloy K. J. & Powrie F. Intestinal homeostasis and its breakdown in inflammatory bowel disease. Nature 474, 298–306 (2011). 21677746 10.1038/nature10208

[b30] Strober W. & Fuss I. J. Proinflammatory cytokines in the pathogenesis of inflammatory bowel diseases. Gastroenterology 140, 1756–1767 (2011). 21530742 10.1053/j.gastro.2011.02.016 PMC3773507

[b31] Ciorba M. A. *et al.* Induction of IDO-1 by immunostimulatory DNA limits severity of experimental colitis. J Immunol. 184, 3907–3916 (2010). 20181893 10.4049/jimmunol.0900291 PMC2945286

[b32] Alex P. *et al.* Distinct cytokine patterns identified from multiplex profiles of murine DSS and TNBS‐induced colitis. Inflamm Bowel Dis. 15, 341–352 (2009). 18942757 10.1002/ibd.20753 PMC2643312

[b33] McAuley J. L. *et al.* MUC1 cell surface mucin is a critical element of the mucosal barrier to infection. J Clin Invest. 117, 2313–2324 (2007). 17641781 10.1172/JCI26705 PMC1913485

[b34] Linden S., Sutton P., Karlsson N., Korolik V. & McGuckin M. Mucins in the mucosal barrier to infection. Mucosal immunol. 1, 183–197 (2008). 19079178 10.1038/mi.2008.5 PMC7100821

[b35] Ueno K. *et al.* MUC1 mucin is a negative regulator of toll-like receptor signaling. Am J Respir Cell Mol Biol. 38, 263–268 (2008). 18079492 10.1165/rcmb.2007-0336RC PMC2258447

[b36] Williams M. A. *et al.* Deletion of the mucin-like molecule muc1 enhances dendritic cell activation in response to toll-like receptor ligands. J Innate Immun. 2, 123–143 (2010). 20375631 10.1159/000254790 PMC2840248

[b37] Mellor A. L. & Munn D. H. IDO expression by dendritic cells: tolerance and tryptophan catabolism. Nat Rev Immunol. 4, 762–774 (2004). 15459668 10.1038/nri1457

[b38] Platten M., Wick W. & Van den Eynde B. J. Tryptophan catabolism in cancer: beyond IDO and tryptophan depletion. Cancer Res. 72, 5435–5440 (2012). 23090118 10.1158/0008-5472.CAN-12-0569

[b39] Klonowski K. *et al.* Inhibition of Indoleamine 2,3-dioxygenase (IDO) enhances the memory CD8 T cell response and modulates the immunodominance hierarchy following influenza infection. J Immunol. 188, 48.17 (2012).

[b40] Blumenthal A. *et al.* M. tuberculosis induces potent activation of IDO-1, but this is not essential for the immunological control of infection. PLoS One. 7, e37314 (2012). 22649518 10.1371/journal.pone.0037314 PMC3359358

[b41] Scott G. N. *et al.* The immunoregulatory enzyme IDO paradoxically drives B cell-mediated autoimmunity. J Immunol. 182, 7509–7517 (2009). 19494274 10.4049/jimmunol.0804328 PMC2741092

[b42] Laroui H. *et al.* Dextran sodium sulfate (DSS) induces colitis in mice by forming nano-lipocomplexes with medium-chain-length fatty acids in the colon. PLoS One. 7, e32084 (2012). 22427817 10.1371/journal.pone.0032084 PMC3302894

[b43] Weigmann B. *et al.* Isolation and subsequent analysis of murine lamina propria mononuclear cells from colonic tissue. Nat Protoc. 2, 2307–2311 (2007). 17947970 10.1038/nprot.2007.315

[b44] Subramanian A. *et al.* Gene set enrichment analysis: a knowledge-based approach for interpreting genome-wide expression profiles. Proc Natl Acad Sci USA. 102, 15545–15550 (2005). 16199517 10.1073/pnas.0506580102 PMC1239896

[b45] Sturn A., Quackenbush J. & Trajanoski Z. Genesis: cluster analysis of microarray data. Bioinformatics 18, 207–208 (2002). 11836235 10.1093/bioinformatics/18.1.207

