# Supplementary material for: Severity of DSS-induced colitis is reduced in Ido1-deficient mice with down-regulation of TLR-MyD88-NF-kB transcriptional networks
Source: Sci Rep. 2015 Nov 27;5:17305. doi: 10.1038/srep17305 (PMC4661522; doi:10.1038/srep17305)
Supplement: Supplementary Information [file srep17305-s1.pdf]

Severity of DSS-induced colitis is reduced in Ido1-deficient mice with down-regulation of TLR-MyD88-NF-kB transcriptional networks

Woo-Jeong Shon[1+]*, Young-Kwan Lee*[2+]*, Ji Hee Shin*[1]*, Eun Young Choi*[2*]*, Dong-Mi Shin*[1,3*]

[1]Department of Food and Nutrition, Seoul National University, Seoul 151-742, Korea

[2]Department of Biomedical Sciences, Graduate School of Seoul National University, Seoul 110-799, Korea

[3]Research institution of human ecology, Seoul National University, Seoul 151-742, Korea

*corresponding authors: shindm@snu.ac.kr; eycii@snu.ac.kr

+these authors contributed equally to this work

Supplementary Figure S1

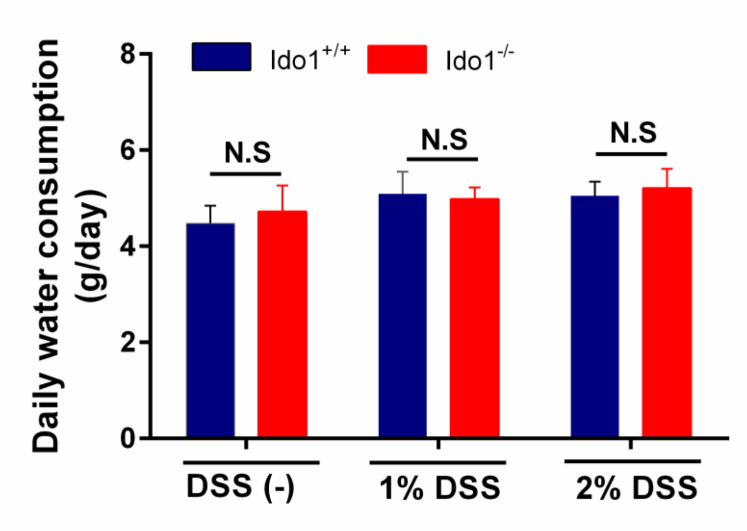

Supplementary Figure S1 | Daily water consumption of *Ido1*$^{-/-}$ and *Ido1*$^{+/+}$ mice. Average water consumption per day per mouse over seven days of DSS treatment was presented. Data are presented as mean ± S.E.M (n = 3-5 per group) and *p* value was estimated by unpaired t test; NS, Not significant.

Supplementary Figure S2

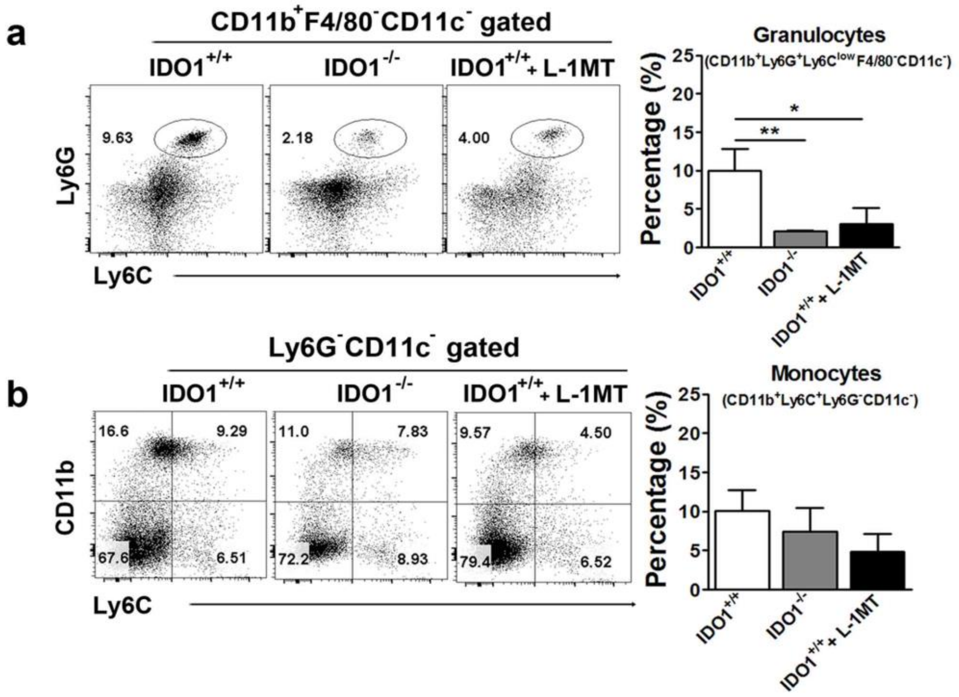

Supplementary Figure S2 | Flow cytometric analysis of the expression of monocytic and granulocytic populations in the presence or absence of IDO1 in DSS induced colitis. Colonic cells in lamina propria were isolated from 2% DSS treated *Ido1*$^{+/+}$, *Ido1*$^{-/-}$ and *Ido1*$^{+/+}$ treated with L-1MT mice on day 7 and 8 after DSS treatment and analyzed difference of the monocytic and granulocytic populations between groups. (a) The expression level of CD11b+Ly6G+Ly6ClowF4/80-CD11c-granulocytes[29] were analyzed using PE-Cy7-conjugated anti-CD11b, eFluor® 450-conjugated anti-Ly6G, APC-conjugated anti-Ly6C, PE-Cy5-conjugated anti-F4/80 and APC-Cy7 conjugated anti-CD11c Abs. (b) The percentage of CD11b+Ly6G-Ly6C+CD11c- monocytic populations[29] were also determined by staining with PE-Cy7-conjugated anti-CD11b, eFluor® 450-conjugated anti-Ly6G, APC-conjugated anti-Ly6C and APC-Cy7 conjugated anti-CD11c Abs. *p < 0.05 and **p < 0.01.
